# Common gas phase molecules from fungi affect seed germination and plant health in *Arabidopsis thaliana*

**DOI:** 10.1186/s13568-014-0053-8

**Published:** 2014-07-15

**Authors:** Richard Hung, Samantha Lee, Cesar Rodriguez-Saona, Joan W Bennett

**Affiliations:** 1 Department of Plant Biology and Pathology, Rutgers, The State University of New Jersey, 59 Dudley Rd., New Brunswick 08901, NJ, USA; 2 Department of Entomology, Rutgers, The State University of New Jersey, 96 Lipman Drive, New Brunswick 08901, NJ, USA

**Keywords:** Volatile organic compound, Arabidopsis thaliana, Fungi, Seed germination, Chlorophyll concentration, Gas chromatography, Mass spectroscopy

## Abstract

Fungal volatile organic compounds (VOCs) play important ecophysiological roles in mediating inter-kingdom signaling with arthropods but less is known about their interactions with plants. In this study, *Arabidopsis thaliana* was used as a model in order to test the physiological effects of 23 common vapor-phase fungal VOCs that included alcohols, aldehydes, ketones, and other chemical classes. After exposure to a shared atmosphere with the 23 individual VOCs for 72 hrs, seeds were assayed for rate of germination and seedling formation; vegetative plants were assayed for fresh weight and chlorophyll concentration. All but five of the VOCs tested (1-decene, 2-n-heptylfuran, nonanal, geosmin and -limonene) had a significant effect in inhibiting either germination, seedling formation or both. Seedling formation was entirely inhibited by exposure to 1-octen-3-one, 2-ethylhexanal, 3-methylbutanal, and butanal. As assayed by a combination of fresh weight and chlorophyll concentration, 2-ethylhexanal had a negative impact on two-week-old vegetative plants. Three other compounds (1-octen-3-ol, 2-ethylhexanal, and 2-heptylfuran) decreased fresh weight alone. Most of the VOCs tested did not change the fresh weight or chlorophyll concentration of vegetative plants. In summary, when tested as single compounds, fungal VOCs affected *A. thaliana* in positive, negative or neutral ways.

## Introduction

Volatiles organic compounds (VOCs) are low molecular mass compounds with high vapor pressure and low to medium water solubility that exist in the gaseous state at room temperature (Herrmann [2010]). Approximately 250 VOCs have been identified from fungi (Chiron and Michelot [2005]) as the products of both primary and secondary metabolism (Turner and Aldridge [1983]; Korpi et al. [2009]). These gas phase molecules are emitted in complex mixtures that vary quantitatively and qualitatively depending not only on the age and genetic profiles of the producing species but also on extrinsic variables such as substrate, temperature, moisture level and pH (Sunesson et al. [1995]; Claeson et al. [2002]; Matysik et al. [2008]). Fungal volatiles have distinctive odorant properties and they have been studied extensively for their positive and negative sensory properties. They impart unique aromas and flavors to mold-ripened cheeses, Japanese koji and other mold-fermented food products (Steinkraus [1983]; Kinderlerer [1989]), and are responsible for the bouquet of gourmet mushrooms such as boletes, chanterelles and truffles (Cho et al. [2008]; Fraatz and Zorn [2010]). On the negative side, when foods are contaminated by molds, they produce off flavors. VOCs have been used as an indirect indicator of fungal spoilage in agricultural products (Borjesson et al. [1992]; Jelen and Wasowicz [1998]; Schnürer et al. [1999]) and of mold contamination in water-damaged buildings (Kuske et al. [2005]; Sahlberg et al. [2013]). Finally, because VOCs can diffuse through the atmosphere and the soil, they are well adapted for signaling between species that share a common ecological niche. Both bacterial and fungal VOCs play competitive roles in chemical interactions between microorganisms (Beattie and Torrey [1986]; Morath et al. [2012]). Many plant and microbial volatile molecules function as semiochemicals, otherwise known as “infochemicals”, and there is a large literature on the ability of fungal VOCs to mediate arthropod behavior, where they have properties as synomones, allomones, and kairomones (Rohlfs et al. [2005]; Mburu et al. [2011]; Davis et al. [2013]). The fungal VOC commonly called “mushroom alcohol” (1-octen-3-ol) is responsible for much of the musty odor associated with mold contamination and is an important insect semiochemical. It attracts many insect species, including the malaria mosquito (Takken and Knols [1999]; Thakeow et al. [2008]).

In contrast, the interactions between fungal VOCs and plants have not received much scientific attention (Bitas et al. [2013]). Based on the observation that there is very little vegetation in areas known to have truffles (subterranean gourmet fungi), it has been hypothesized that these fungi may have the ability to suppress plant growth through their volatiles (Splivallo et al. [2007]). Kishimoto et al. ([2007]) have shown that 1-octen-3-ol enhances resistance of mature plants of *Arabidopsis thaliana* to *Botrytis cinerea* and activates some of the same defense genes turned on by ethylene and jasmonic acid signaling, important plant hormones involved in plant defense.

We hypothesized that we could distinguish between bioactive and inactive fungal vapors by using chemical standards of individual VOCs and then exposing plants to controlled concentrations in a model habitat. We selected *A. thaliana* as our test species due to the many benefits associated with the use of a well recognized model system including but not limited to: small size, short life cycle, genetic tractability, and comprehensively researched background. Preliminary studies have also shown that tomato plants exposed to VOCs are affected in a similar fashion to *A. thaliana* exposed to the same VOCs indicating that *A. thaliana* is a good model organism for study. Similarly, the effects of fungal VOCs on plant development have been demonstrated in several plants in *Brassicaceae* family including radish, cabbage, rape, and broccoli (Ogura et al. [2000]). The aim of our study was to evaluate the effect of individual fungal VOCs on seed germination, vegetative plant growth and chlorophyll concentration in a controlled environment. In this report, our specific objectives have been to create standardized model exposure habitats in order to compare the possible stimulatory and inhibitory effects of fungal VOCs from different chemical classes (e.g., alcohols, aldehydes, ketones, and so forth) and to conduct exposure studies using *A. thaliana* seeds and two-week-old vegetative plants.

## Materials and methods

### Plant material and seed preparation

All volatile exposure tests were done with *Arabidopsis thaliana* ecotype Columbia 7. Surface-sterilization of seeds and seedling formation studies were conducted as described previously with slight modifications (Hung et al. [2013]). Surface sterilized seeds were sown on Murashige and Skoog (MS) media with vitamins, 3% sucrose, and 0.3% Gellan Gum Powder (G 434 PhytoTechnology Laboratories, Shawnee Mission, KS). In germination-seedling formation studies, seeds were sown on Petri dishes (20 seeds per plate) with 20 ml MS media and placed at 4°C in the dark for three days to stratify the seeds. Seeds used to grow plants for exposure assays of two–week old plants were sown individually in test tubes with 10 ml of MS, covered with plant tissue culture caps and then stratified as described above. After three days, the stratified seeds in their individual test tubes were placed in a growth chamber at 21°C ± 2°C with a 16 hour photoperiod for two weeks prior to exposure to VOCs.

### Chemicals and exposure conditions

Authentic standards of these high purity chemicals were purchased in liquid form from Sigma-Aldrich (St. Louis, Missouri). The criteria for the selection of these compounds were: 1) the volatiles should represent different chemical classes, 2) they had been isolated from a range of fungal species including both mushrooms and molds, and 3) that they included several VOCs commonly found in soils.

The germination and vegetative exposure to VOCs were determined using the methods described previously (Hung et al. [2014]; Lee et al. [2014]). Seeds (in Petri plates) or two-week-old plants (in individual test tubes) were exposed in one liter culture vessels (see Additional file 1). All tests were done at a low concentration similar to the concentration of VOCs analyzed previously: one part per million (1 ppm = 1 μl/l). The desired concentration of 1 ppm in the test container was obtained by depositing a drop of the chemical standard (VOC) in liquid form onto the inside of the glass vessel. The compounds, due to their chemical properties will quickly volatilize into the gas phase in the test conditions. Before sealing the lids, a 10 × 10 cm piece of Dura Seal Cling Sealing Film (Diversified Biotech) was placed over the top of each culture vessel so as to prevent VOC leakage through the polypropylene closure. The culture vessels containing either seeds in Petri plates or two-week-old plants in test tubes were arranged randomly in the growth chamber and then placed on a one inch throw rotator at 40 rpm in order to volatilize and evenly distribute the compounds. The control plants were placed in identical conditions without any VOCs.

### Scoring germination stages

The seeds were exposed to the individual VOCs for 72 hours and then examined under a binocular microscope where they were scored into three categories: no germination, germination (emergence of the radical [embryonic root]), and seedling formation (presence of the radicle, the hypocotyls and the cotyledons) (see Additional file 2). Seeds scored as “no germination” included seeds with a ruptured testa (seed coat) but without the presence of the radicle.

### Plant mass and chlorophyll concentration

After exposure to the vapors of the individual VOCs, plants were removed from the test conditions, the shoot and leaves were cut away from the roots, and fresh weight of the shoots and leaves was obtained. The chlorophyll was extracted using the method of Jing et al. ([2002]) with some modifications. The plants were soaked overnight in 80% acetone at 4˚C in darkness prior to obtaining photometric readings at 663 and 645 nm with a spectrophotometer (DU800, Beckman Coulter, Brea, CA). Total chlorophyll = 20.2 (A_645_) + 8.02 (A_663_)(V/1,000 * w) where V = total volume of the sample, w = weight of the sample, A_663_ = absorbance at 663 nm, A_645_ = absorbance at 645 nm (Palta [1990]). Each solvent extract contained one plant per treatment.

### Statistical analysis

The data were analyzed and plotted using Excel software (Microsoft, Redmond, WA) and SigmaPlot (SPSS Science Inc., IL). To test the significance of the exposure studies, one-way analysis of variance (ANOVA) and Student’s t-tests were performed with the aggregated data. The Student’s t-tests determine if there are significant differences between two sets of data: the control and VOC exposed plants. For germination exposures, two replicate plates with 20 seeds per plate were tested for each compound, with two independent experiments, for a total of 80 seeds. For vegetative plant exposure, four plants were placed in the exposure vessel and three jars were used for each experiment. There were three independent experiments for each compound, for a total of 36 plants.

## Results

### Seedling formation tests

**Figure 1:**
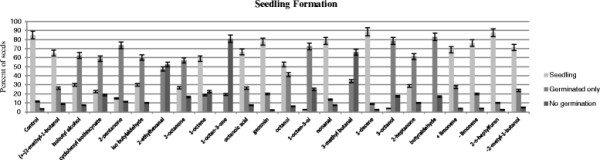
**Percentage of seeds that have reached each stage after 72 hrs of exposure to 23 fungal VOCs.** Standard error indicated in error bars.

**Figure 2:**
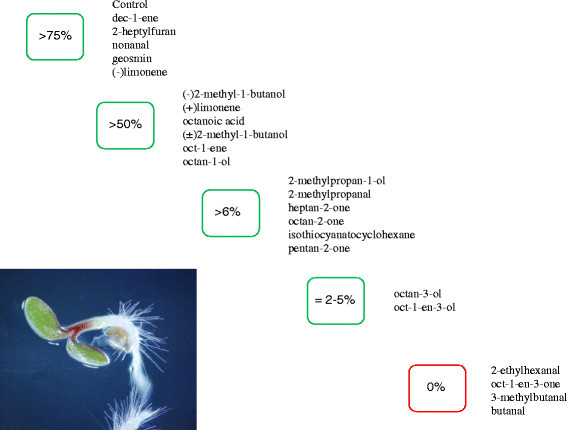
**A summary of the categories of percent seedling formation of** ***A. thaliana*** **seeds after 72 hrs of exposure to chemical standards of 23 different volatile organic compounds.**

The percentage of seeds germinating and progressing to seedling formation after exposure to the 23 fungal volatiles is shown in Figure 1 and summarized in Figure 2. In our experiments, more than 75% of control seeds progressed to the seedling stage after 72 hrs. Similar rates of seedling formation were observed for seeds exposed to 1-decene, 2-n-heptylfuran, nonanal, geosmin and -limonene. In contrast, none of the seeds exposed to 2-ethylhexanal 1-octen-3-one, 3-methylbutanal, or butanal formed seedlings. Of these four inhibitory volatiles, radical protrusion (i.e. germination) was observed in only 19% of 1-octen-3-one exposed seeds, while 83% of seeds exposed to butanal exhibited formation of a radical. Seeds exposed to the other 14 volatile compounds tested had intermediate levels of germination efficiency and seedling formation (Figure 2). Of the five aldehydes we tested, three (2-ethylhexanal, 3-methylbutanal, or butanal) inhibited seedling formation by 100% and one (3-methylproponal) inhibited seedling formation by 70%. Nevertheless, no matter the extent of inhibition in the presence of the 23 VOCs tested, when removed from exposure to the VOCs after 72 hr, all seeds resumed germination and formed seedlings.

### Vegetative plants tests

**Figure 3:**
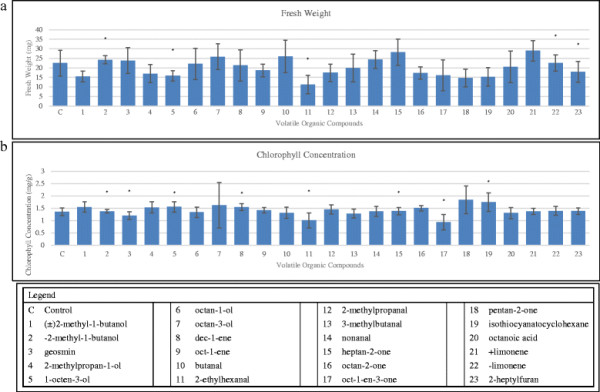
**Fresh weight and chlorophyll concentration of two week old plants of *A. thaliana*** **exposed for 72 hrs to chemical standards of 23 different fungal volatile organic compounds.a**. Fresh weight in mg. **b**. Chlorophyll concentration in mg/g. Significant values compared to control are marked with an asterisk p < 0.04. Standard error indicated in error bars.

The fresh weight and chlorophyll concentration of control and exposed plants are given in Figure 3. The fresh weight of control plants was 22.4 mg (±6.7 SE). A significant decrease in fresh weight was observed after 72 hr exposure to 1-octen-3-ol, 2-ethylhexanal, and 2-heptylfuran, where the mean fresh weight was respectively 6.7 mg (±2.7 SE), 11.2 mg (±4.9 SE), and 6.3 mg (±5.4 SE) less than controls. The other compounds tested ((±)2-methyl-1-butanol, geosmin, 2-methylpropan-1-ol, 1-octen-3-ol, octan-1-ol, octan-3-ol, dec-1-ene, oct-1-ene, butanal, 2-ethylhexanal, 2-methylpropanal, 3-methylbutanal, nonanal, heptan-2-one, octan-2-one, oct-1-en-3-one, pentan-2-one, isothiocyanatocyclohexane, octanoic acid, +limonene, −limonene, and 2-heptylfuran) did not cause significant differences in fresh weight of exposed vegetative plants (Figure 3).

In Figure 3, we have expressed the chlorophyll concentration data as mg/gm of fresh weight of shoots and leaves. Five compounds (−2-methyl-1-butanol, 1-octen-3-ol, dec-1-ene, heptan-2-one, and isothiocyanatocyclohexane) yielded statistically significant increases in chlorophyll concentration showing, respectively, 0.02 mg/g, 0.2 mg/g, 0.18 mg/g, 0.02 mg/g, and 0.4 mg/g greater amounts than controls. Three compounds, geosmin, 2-ethylhexanal, and 1-octen-3-one caused a statistically significant decrease in chlorophyll concentration of, respectively, 0.15 mg/g, 0.35 mg/g, and 0.42 mg/g less than controls. While geosmin did not adversely affect fresh weight, it did cause a significant decrease in chlorophyll concentration as indicated above. The compound 2-ethyl-hexanal decreased both fresh weight and chlorophyll concentration. The nonracemic form of −2-methyl-1-butanol showed a small but significantly different increase in both parameters.

In conclusion, all but five of the VOCs tested had a significant effect in inhibiting either germination, seedling formation or both. Three of the compounds (2-ethyl-hexanal, 1-octen-3-one, and 3-methylbutanal) showed more than 50% inhibition of seed germination, while 12 of the compounds (2-methylpropan-1-ol, 2-methylpropanal, heptan-2-one, octan-2-one, isothiocyanatocyclohexane, pentan-2-one, octan-3-ol, 1-octen-3-ol, 2-ethylhexanal, 1-octen-3-one, 3-methylbutanal, and butanal) were associated with more than a 50% retardation in seedling formation. Butanal was unusual in that 83% of seeds germinated (i.e. formed a radicle) but none of these germinated seeds progressed to seedling formation. In general, the most bioactive of the VOCs we tested were aldehydes and ketones. The single, most inhibitory VOC against germination and seedling formation was oct-1-en-3-one, an eight carbon ketone that has been isolated from both molds and mushrooms (Jelen and Wasowicz [1998]). Nevertheless, in all cases, VOC exposed seeds were able to resume germination and progress to seedling stage when removed from the shared atmosphere with the VOC. We conclude that the fungal VOCs we tested have a phytostatic (inhibitory), not a phytocidal (lethal) effect on seeds.

## Discussion

In order to study the influence of individual fungal VOCs on plant health, we used *A. thaliana* as our test organism and developed standardized protocols for exposing seeds and young vegetative plants. We investigated a representative sample of common fungal VOCs encompassing seven alcohols, two alkenes, five aldehydes, four ketones, and a single representative isothiocyanate, carboxylic acid, and furan. In addition, we tested both isomers of the terpene 1-methyl-4-(1-methyetenyl)-cyclohexene, commonly known as limonene. We used a 72 hour exposure period, and exposed either seeds or young vegetative plants to 1 ppm of chemical standards of the 23 fungal VOCs in a contained chamber.

Seed germination assays with lettuce, cucumber and other economically important species have been widely employed as low-cost, ethically acceptable toxicity tests to screen for dangerous levels of industrial contamination in water and soils (Banks and Schultz [2005]; Wang et al. [2001]). Almost all of these assay studies have involved aqueous phase compounds, although there have been a few scattered reports describing inhibitory effect of plant volatiles on the germination of seeds from crop and weed species (Holm [1972]; Bradow and Connick [1990]). The physiological basis for this inhibitory action has not been elucidated. *A. thaliana*, though not agriculturally important, offers a number of experimental advantages for studying basic aspects of plant biology, including seed germination, because of its many genetic resources (Koornneef et al. [2002]). Our development of an *A. thaliana* exposure system for studying VOC effects under controlled conditions offers the promise of being able to use this species to dissect the germination inhibition response at the molecular level.

In contrast to seed germination studies, which all report inhibition of germination by VOCs (Holm [1972]; French et al. [1975]; Bradow and Connick [1990]). To date, published studies of the effects of VOCs on vegetative plants report VOC--associated growth stimulation by mixtures of VOCs emitted by growing bacteria or fungi (Ryu et al. [2003]; Minerdi et al. [2011]; Hung et al. [2013]; Paul and Park [2013]). Many soil dwelling microbes emit VOCs that mediate various chemical “conversations” between the rhizosphere and plants (Wenke et al. [2010], [2012]). For example, plant growth promoting rhizobacteria (PGPR) produce mixtures of VOCs that enhance growth in a wide variety of species (Farag et al. [2006]; Vespermann et al. [2007]; Lugtenberg and Kamilova [2009]). In some cases, microbial VOCs induce systemic resistance (Van Loon et al. [1998]; Ryu et al. [2003]) or inhibit the growth of plant pathogens (Minerdi et al. [2009], [2011]). Volatiles of *Cladosporium cladosporioides* enhance growth of tobacco plants (Paul and Park [2013]) and our laboratory has shown that vegetative plants of *A. thaliana* seedlings grown in a shared atmosphere with volatiles emitted by living cultures of the biocontrol fungus *Trichoderma viride*, displayed increased size and vigor (Hung et al. [2013]). Fungal VOCs may contribute to the ability of certain species to outcompete neighboring plants. For example, the VOCs produced by *Muscodor yucantanensis* were toxic to the roots, and inhibited seed germination, of amaranth, tomato and barnyard grass (Macias-Rubalcava et al*.*[2010]). In all of these cases, the growth-enhancing effects were mediated by mixtures of naturally emitted VOCs that change with both growth phase and extrinsic environmental parameters.

In our studies, we used a controlled system and exposed plants to low concentrations of individual VOCs. In this controlled habitat, the VOCs tested either had neutral or negative effects on vegetative plant growth, suggesting that the known growth enhancing effects of bacterial and fungal VOCs maybe by synergistic mixtures working in concert. A parallel example is provided by the antibiotic effects of volatiles produced by *Muscodor albus*. This species produces a mixture of VOCs that inhibit and kill a wide range of plant pathogenic fungi and bacteria (Strobel et al. [2001]). Nevertheless, when *Muscodor* VOCs were tested individually, the inhibitory effects were not observed, suggesting that the antifungal activity required a suite of VOCs working in concert (Strobel et al. [2001]).

It should be recognized that studies on transkingdom signalling mediated by fungal VOCs are technically difficult to conduct. Biogenic VOCs exhibit enormous heterogeneity chemically, spatially, and temporally; are found in low concentrations; and by definition have innate evaporative properties that make it difficult to investigate their impact on plant growth and development in natural settings. Our exploratory research shows that by using controlled concentrations of pure synthetic compounds in model habitats, individual VOCs can be studied one by one, thereby isolating their distinct growth-promoting, growth-inhibiting and other physiological effects. The genetic and genomic resources available for *A. thaliana* make this organism well suited for future research on the mechanistic basis of VOC mediated interactions and for analysis of the consequent biological responses*.* In summary, the study of gas phase fungal metabolites offers many interesting prospects for enlarging our understanding of the way in which fungi interact with plants in nature and may have potential for commercial application of VOCs in greenhouse agriculture.

## Competing interests

The authors declare that they have no competing interests.

## Authors’ contributions

RH designed the volatile exposure studies. In addition, he participated in the volatile exposure experiments and the drafting of the paper. SL participated in the exposure experiments and the drafting of the paper. CR-S collected and analyzed the volatile organic compounds. JWB conceived the study and participated in the drafting of the paper. All authors read and approved the final manuscript.

## Additional files

## Supplementary Material

Additional file 1: Diagrams of exposure chambers for a. seeds in Petri dishes and b. plants in test tubes. Click here for file

Additional file 2: Representative images of stages of seedling formation A) No germination B) Germination, radical protrusion C) Seedling formation. Click here for file
