# Supplementary material for: Common gas phase molecules from fungi affect seed germination and plant health in Arabidopsis thaliana
Source: AMB Express. 2014 Jul 15;4:53. doi: 10.1186/s13568-014-0053-8 (PMC4100562; doi:10.1186/s13568-014-0053-8)
Supplement: Additional file 1: — Diagrams of exposure chambers for a. seeds in Petri dishes and b. plants in test tubes. [file s13568-014-0053-8-S1.doc]

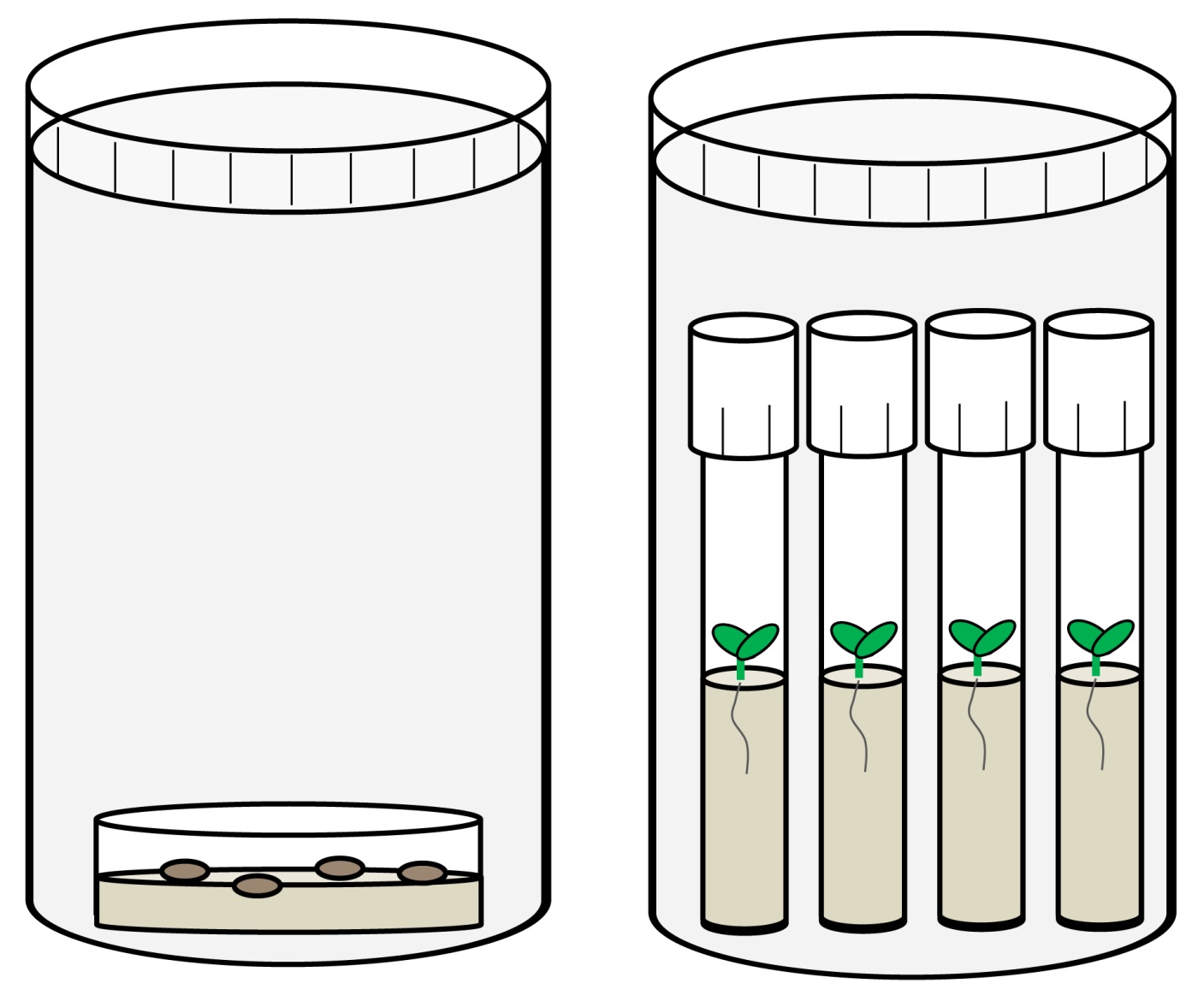


Additional file 1. Diagrams of exposure chambers for a. seeds in Petri dishes and b. plants in test tubes

a

b
