# Supplementary material for: Common gas phase molecules from fungi affect seed germination and plant health in Arabidopsis thaliana
Source: AMB Express. 2014 Jul 15;4:53. doi: 10.1186/s13568-014-0053-8 (PMC4100562; doi:10.1186/s13568-014-0053-8)
Supplement: Additional file 2: — Representative images of stages of seedling formation A) No germination B) Germination, radical protrusion C) Seedling formation. [file s13568-014-0053-8-S2.doc]

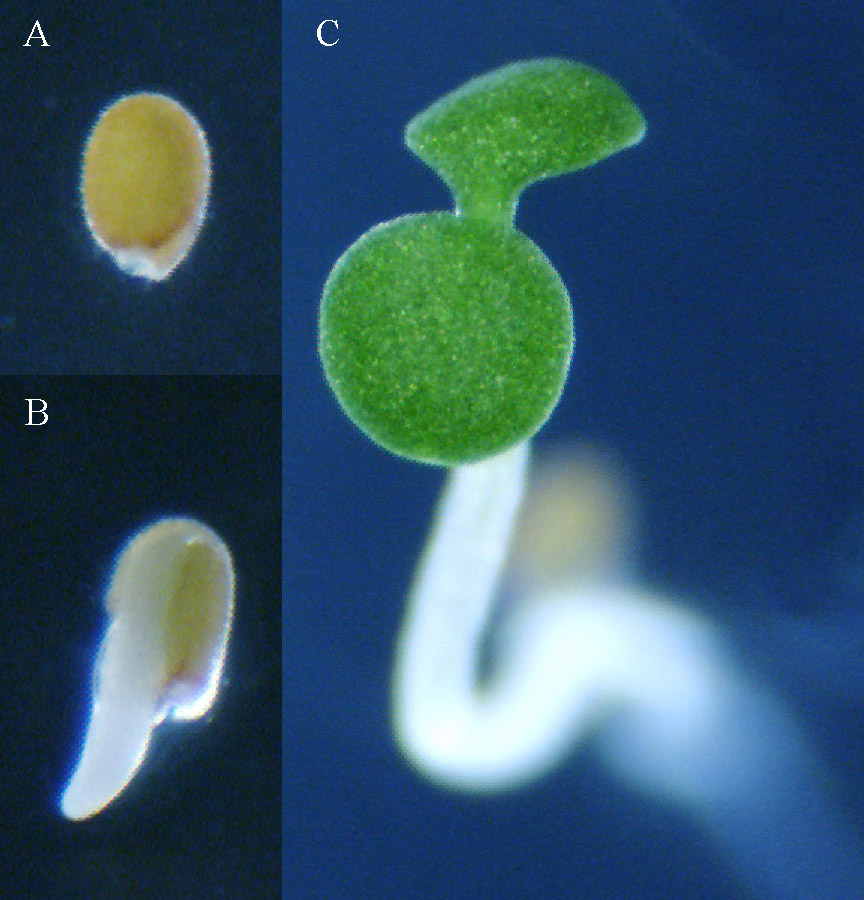


Additional file 2. Representative images of stages of seedling formation A) No germination B) Germination, radical protrusion C) Seedling formation
